# Does Cognitive Training Reduce Falls across Ten Years?: Data from the ACTIVE Trial

**DOI:** 10.3390/ijerph20064941

**Published:** 2023-03-11

**Authors:** Briana N. Sprague, Lesley A. Ross, Karlene K. Ball

**Affiliations:** 1 Division of General Internal Medicine & Geriatrics, University of Indiana School of Medicine, Indianapolis, IN 46202, USA; 2 Center for Aging Research, Regenstrief Institute, Indianapolis, IN 46202, USA; 3 Department of Psychology, Clemson University, Clemson, SC 29634, USA; 4 Department of Psychology, University of Alabama at Birmingham, Birmingham, AL 35233, USA

**Keywords:** falls, speed of processing training, memory training, reasoning training, long-term follow-up

## Abstract

The purpose of this study was to examine the effect of cognitive training on the risk of experiencing a fall across 10 years. The study used data from the Advanced Cognitive Training for Independent and Vital Elderly (ACTIVE) randomized controlled trial. Older adults aged 65–94 were randomly assigned to speed of processing, memory, or reasoning training or to a no-contact control group (*n* = 2802). The experience of a fall in the prior two months was assessed at baseline and at 1, 2, 3, 5, and 10 years posttest. Cox proportional hazards explored group differences in the total sample, as well as group differences for participants classified as low risk (*n* = 2360) and high risk (*n* = 442) for future falls. The data were censored at the first reported fall postbaseline. After baseline, 983 (35.08%) participants across the full sample reported a fall. There were no significant effects of the training in the full sample or in the low-risk sample of participants. However, the participants at greater risk for future falls in the speed of processing training group were 31% less likely (HR = 0.69; 95% CI = 0.48, 0.998, *p* = 0.049) to experience a subsequent fall across ten years compared to the control group. Reasoning and memory training did not reduce a future fall in the high-risk sample. The speed of processing training reduced the risk of future falls across ten years in the high-risk participants. Future work should examine moderators and mediators of training in at-risk samples.

## 1. Introduction

Falling is common, costly, and the leading cause of nonfatal injury for older adults in the US [1]. Older adults have increased injury and mortality after falling for several reasons, including skeletal fragility, fracture risk [2], and comorbidities that reduce the likelihood of recovery [3]. Even after noninjurious falls, older adults are likelier to experience recurrent falls [4] and increased fear of falling [5], as well as reduced physical activity engagement [6]. This is problematic because the fear of falling [5] predicts subsequent falls, leading to a cycle of excessive disability. Thus, a previous fall may put older adults at even greater risk for subsequent falls. Identifying alternative programs that do not rely on physical activity to reduce the risk of falling is critical for the large proportion of older adults who have experienced or are afraid of falling.

Physical activity interventions currently have the strongest evidence for falls reduction [7] through improving measures of complex physical function associated with falls, e.g., balance and lower limb strength [8]. Older adults also cite physical activity as being effective for preventing falls, but this knowledge does not necessarily transfer to behavior modification or a reduction in falls [8]. For instance, older adults who experienced a fall either do not change or actually reduce their physical activity engagement postfall [6]. Most older adults with physical limitations can engage in physical activity interventions [9], but they may be disinclined to participate due to various barriers, including fear of falling or injury. Because of these barriers and limitations of physical activity interventions, researchers are increasingly highlighting the need for alternative, complementary, and nonphysical activity interventions [10,11].

A growing body of experimental evidence demonstrates that cognitive training can have positive transfer effects on complex physical functions, a correlate of falls, in older adults [10,11,12,13,14,15,16,17,18,19,20]. In particular, speed of processing training shows promise in maintaining or attenuating age-related decline in complex physical function, and emerging evidence suggests reasoning training may also confer benefits to complex physical functions as well [10]. The degree to which cognitive training directly impacts falls is unknown; however, given the repeated transfer effects of some process-based cognitive training programs to physical functioning, it is reasonable to assume that there may be positive impacts to other lower limb-related outcomes such as falls.

While some cognitive training programs, especially speed of processing (also known as divided attention and useful field of view training), have the potential to positively impact falls, such training may not be equally effective across all individuals. Samples at increased risk for future falls and other mobility outcomes may receive greater benefits from some cognitive training programs across a breadth of outcome measures, including physical function [11], speed of processing [21,22], driving mobility and cessation [23,24,25,26], and everyday functions [21,27]. Coupled with the increased interest in identifying moderators of cognitive training transfer, this suggests that it is critical to consider whether training effects are greater among those at greater risk for functional decline. In the case of falls, experiencing a prior fall is one of the most robust predictors of future falls [4]. Thus, participants reporting a history of falls within the prior two months of baseline were considered at greater risk compared to participants without such a report.

The goal of the current study was to examine the effect of three cognitive training programs on the time to the first reported fall across 10 years in a sample of community-dwelling older adults. Based on prior research on the impact of cognitive training programs on complex physical functions, we hypothesized that cognitive training, particularly the speed of processing and reasoning training arms, would be associated with a delay in the time to a first reported fall after controlling for complex physical function and demographic covariates. Additionally, given the importance of baseline risk status as a moderator of transfer effects [11,21,22,23,25,27], we hypothesized that participants at greater risk for future falls would benefit the most from cognitive training, specifically speed of processing and reasoning training.

## 2. Methods

### 2.1. Participants

This study used secondary data from the Advanced Cognitive Training for Independent and Vital Elderly (ACTIVE) study, a multisite, randomized clinical trial investigating the effect of three cognitive training programs on health and functional outcomes across a 10-year period. Falls were an a priori identified secondary outcome of the ACTIVE trial, but current analyses were not preregistered. The inclusion criteria were age 65 or older, visual acuity ≥ 20/50, Mini-Mental State Examination score ≥ 23, no health conditions associated with cognitive impairment, verbal communication skills, no difficulties performing basic activities of daily living, and no recent participation in cognitive training. Thirty participants were removed due to the fact of improper randomization, leaving a sample of 2802 older adults. At baseline, the participants were on average 73.64 years old (SD = 5.91), predominantly women (*n* = 2121; 75.83%), predominantly White (*n* = 2035, 72.80%), and reported an average of 13.53 years of education (SD = 2.08). The sample’s descriptive statistics for each arm and the full sample are presented in Table 1. Extensive details about ACTIVE can be found elsewhere [28], including using ClinicalTrials.gov Identifier NCT00298558. Informed consent followed the requirements of each research institution’s human subjects review committee. The research institutions included Pennsylvania State University, the University of Alabama at Birmingham, Indiana University, The Hebrew Rehabilitation Center for Aged in Boston, Johns Hopkins University, and Wayne State University. The recruitment methods varied by site and included the efforts of local churches, senior or other community centers, housing sites, and eye clinics. More details on the site-specific recruitment methods can be found elsewhere [28]. Consent to participate was obtained three times: during the telephone screening (verbal consent), in-person assessment (written consent), and completion of baseline assessment/full-study participation prior to randomization (written consent). The testers but not the participants themselves were blinded to the group allocation.

### 2.2. Study Design and Procedures

The baseline assessments for the ACTIVE study were conducted between March 1998 and October 1999, and a 10-year follow-up was completed in December 2010. Prior to randomization, baseline cognitive and physical functions, lifestyle factors, and health assessments were conducted. The initial cognitive training interventions consisted of ten 60-to-75 min, trainer-led sessions administered over six weeks conducted in group settings. All intervention materials were developed by the research team. The follow-up assessments were conducted approximately two months (i.e., posttest), one year, two years, three years, five years, and 10 years after baseline testing. The compliant training arm participants (i.e., completed at least 8 of 10 sessions) were further randomized to either receive four booster sessions prior to the assessments in years one and three (totaling 8 possible booster sessions) or received no booster training.

#### 2.2.1. Speed of Processing Training Arm

This was process- and computer-based training that focused on improving the display speeds at which participants could correctly identify increasingly complex visual information. The training comprised four tasks. For each task, the score was the shortest presentation time needed to perform the task correctly 75% of the time. The four tasks reflected increments in difficulty, from a simple identification task in which the participants determined which of two objects (car or truck) appeared in a fixation box up to a complex task in which the participants judged which configuration of objects (two cars, two trucks, or a car and truck) appeared in a fixation box in the middle of a screen while simultaneously identifying the location of a peripheral target on the outside of a cluttered display [28].

#### 2.2.2. Memory Training Arm

This was a strategy-based pencil-and-paper training designed to improve verbal episodic memory using mnemonic strategies. The training included practice and feedback in organizing materials into meaningful categories, such as lists of errands or grocery shopping. After a brief introduction and overview of the main memory ability targeted by the training session, the participants received instruction in a strategy or mnemonic rule, exercises to practice the rule, individual and group feedback on performance, self-efficacy enhancement, and a practice test. In the strategy instruction, the trainer described the rule or strategy in relation to four basic memory principles (meaningfulness, organization, visualization, and association) and modeled the strategy’s use with concrete examples. The participants practiced the strategy during both the individual and group exercises involving lab-like memory tasks, as well as memory tasks related to the activities of daily life [28].

#### 2.2.3. Reasoning Training Arm

This was a strategy-based pencil-and-paper training that focused on improving problem solving and included practice and feedback on identifying patterns or sequences. The participants were given opportunities to practice the strategies in both individual and group exercises. The exercises involved abstract reasoning tasks (e.g., letter series), as well as reasoning problems related to activities of daily life (e.g., identifying medication dosing patterns and filling in a pill reminder case [28]).

#### 2.2.4. No-Contact Control Arm

The participants randomized to the no-contact control arm came to the study site for all assessments, and no intervention was conducted.

### 2.3. Measures

#### 2.3.1. Training

**Figure 1:**
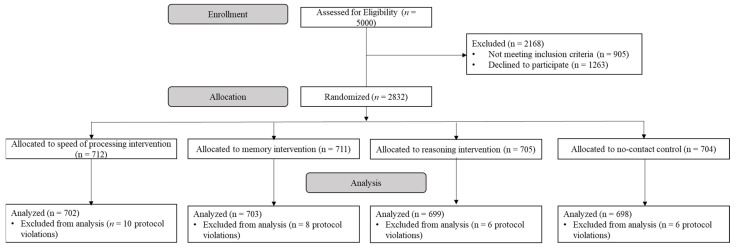
CONSORT flow chart of the ACTIVE trial.

The intention-to-treat (ITT) models assessed the effect of randomized training regardless of adherence. The training groups were separately compared against the no-contact control group and in some cases against each other. The follow-up analyses included those assigned to further booster sessions. See Figure 1 for the CONSORT flow chart of the ACTIVE trial.

#### 2.3.2. Falls

The participants were asked whether they had experienced a fall (0 = no, 1 = yes), defined as instances of accidentally losing their balance and falling on the ground or against an object such as furniture in the prior two months as part of a self-administered questionnaire. Additional efforts to verify a fall experience were not conducted. The falls were assessed at baseline and again at 1, 2, 3, 5, and 10 years posttest. The primary analyses predicted the likelihood of prospective falling for all participants. Subgroup analyses were also conducted on those with a higher baseline risk of falling, defined as those who experienced a fall in the two months prior to baseline.

#### 2.3.3. Complex Physical Function

Turn 360, a measure of complex lower extremity function and balance, was included as a functional confounder [29,30], as this domain has been shown to be related to both falls [31] and cognitive function [32]. From a standing position, the participants were asked to turn in a complete circle as quickly and safely as possible. Walking aids were permitted for use in the Turn 360 protocol. The average of two trials (or one if only completed once) at baseline was included; higher scores indicated more steps (i.e., worse performance).

#### 2.3.4. Demographic Covariates

Age at baseline, gender, race (White vs. Black), educational attainment, and baseline cognitive status assessed by the Mini-Mental Status Examination [33] were included as covariates.

### 2.4. Analytic Strategy

The ACTIVE trial was powered to detect the effect sizes of 0.20 based on the observed effect sizes of previous cognitive training research at the time [28]. Given the baseline proportion of falls (0.158) and the sample size per group, we were adequately powered to detect effect sizes as small as *d* = 0.10.

Chi-square and ANOVAs were used to test the baseline demographics and Turn 360 differences by baseline fall status. For the Cox regressions, time was right censored and calculated as the number of months between the baseline assessment and the 10-year follow-up for persons who did not report a fall within 10 years. For individuals who either dropped out of the study or experienced a fall, the date of their last assessment or time when a first fall was recorded was used to define the time event, respectively. If participants missed one follow-up session but completed sessions afterward (e.g., a participant missed Annual 1 but completed Annuals 2–10), the date of their last assessment or first reported fall was the time-censored event. Then, base Cox regression models were used to assess the probability that an individual would experience a fall after controlling for baseline Turn 360 and significant demographic covariates using the control group. Then, four sets of models assessing the impact of the cognitive training on future falls were conducted. First, the intention to treat analyses examined the relationship between randomization to training (i.e., memory, reasoning, or speed of processing compared to the no-contact control condition) and time until a future fall. Second, this model was repeated with only participants who received booster training to assess if increased training, via booster, impacted the time until a future fall. Finally, the relationship between randomization to cognitive training and time to a future fall was examined as a function of baseline risk, such that models were examined separately among participants with low (no falls reported prior to baseline) and high (a fall reported prior to baseline) risk of future falls. All data analyses were conducted with SPSS, version 26 (IBM Corporation, Armonk, NY, USA), and significance was considered as *p* < 0.05 for two-tailed tests.

## 3. Results

At baseline, 442 participants (15.82% of the total sample; *n* = 2793) reported experiencing a fall in the prior two months. In total, 983 (35.08% of the full sample) reported a fall over the 10-year follow-up period. The average length of time from baseline to the first reported fall was 40.67 months (3.39 years; SD = 33.68 months, or 2.81 years).

The results of the base model in the control group revealed that poorer performance on the Turn 360 task (HR = 1.10, 95% CI = 1.03, 1.18, *p* = 0.01 was associated with a higher risk of a future reported fall. The baseline age (HR = 1.01, 95% CI = 0.98, 1.03, *p* = 0.63), gender (HR = 0.92, 95% CI = 0.67, 1.25, *p* = 0.58), race (HR = 1.25, 95% CI = 0.91, 1.71, *p* = 0.17), education (HR = 1.00, 95% CI = 0.95, 1.05, *p* = 0.98), and MMSE score (HR = 1.00, 95% CI = 0.94, 1.08, *p* = 0.91) were not associated with the risk of a future fall and were removed from subsequent analyses.

In the full sample’s ITT analyses, randomization to speed of processing (HR = 0.91, 95% CI = 0.76, 1.09, *p* = 0.31), memory (HR = 0.88, 95% CI = 0.74, 1.06, *p* = 0.19), or reasoning training assignment (HR = 1.00, 95% CI = 0.83, 1.19, *p* = 0.96) was not associated with the time until a future fall across the study period. The same pattern occurred when constraining the ITT analyses to those assigned to receive additional booster sessions (Table 2).

**Figure 2:**
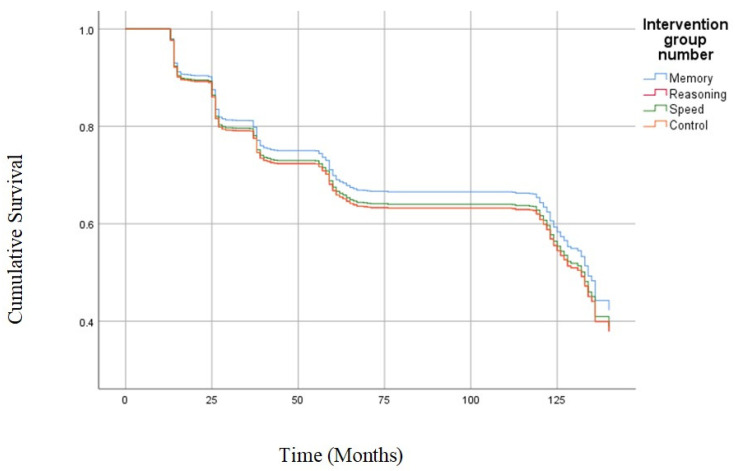
Survival curves of the low-risk sample. There were no significant differences between speed of processing, memory, or reasoning training with the control group and the low-risk sample (*p* > 0.05).

**Figure 3:**
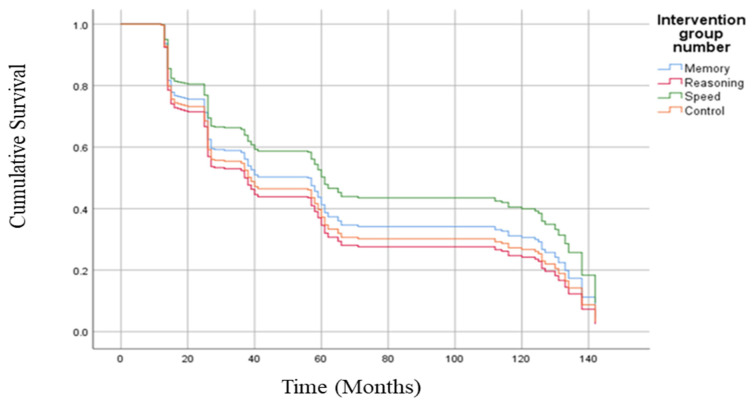
Survival curves of high-risk sample. There were significant differences between the speed of processing and no-contact control group (HR = 0.69, *p* = 0.049) but not the memory or reasoning training groups among the high-risk sample (*p* > 0.05).

Next, we examined the impact of the three cognitive training programs on both the lower and higher risk of future falls using the reported baseline falls as the grouping variable. The cognitive training was not significantly associated with a reduction in the risk of future falls in the low-risk subsample (Figure 2). However, the speed of processing reduced risk of falls approximately 30% across the ten years (HR = 0.69, 95% CI = 0.48, 0.998, *p* = 0.049; Figure 3) in participants with a higher risk of falls. Memory and reasoning training were not associated with the likelihood of experiencing a fall across the study period in the high-risk fall group. Due to the small sample size, we were unable to complete subsample analyses for those receiving booster training.

## 4. Discussion

This was the first study to examine the effect of cognitive training on the time to a reported fall across a 10-year period. When examining the full sample, cognitive training was not associated with the reduced risk for future falls, failing to support our first hypothesis. When examining the participants at greater risk for future falls, the speed of processing training resulted in a 31% reduction of falls across ten years, thus partially supporting our second hypothesis. Consistent with prior work [11,21,22,23,24,25,26,27], these results highlight the importance of examining moderators of training, or the “who benefits” question regarding transfer effects.

Cognitive training in general may not be effective for delaying falls in a healthy, community-dwelling sample, but the speed of processing training may be effective for those with a greater risk for future falls. The speed of processing training generally positively impacts complex lower limb function and balance [10,11,12,13,14,15,16,17,18,19,20], and there is emerging evidence that it is particularly effective for those at greater risk for mobility limitations. For instance, Smith-Ray and colleagues found that Posit Insight training (which included a commercialized version of the speed of processing training) was effective for attenuating declines in gait speed among those with slower baseline gait speed [11]. The results from the current study extend prior work by suggesting that the benefits of the speed of processing training to physical functional outcomes extends to falls. We found an approximately 30% reduction in falls among this subsample, which is comparable to the effectiveness of other fall prevention programs [8]. The mechanisms by which poorer baseline performance are associated with greater training gains are largely unknown, but preliminary evidence suggests that training-related gains in speed of processing [21,22,27] may confer benefits to physical and everyday functional domains that incorporate speed and attentional elements. While mobility is largely physical, walking safely in one’s environment necessitates maintaining speed while simultaneously monitoring for intrinsic (e.g., balance) and extrinsic (e.g., tripping hazards such as a rug) falling factors and are cognitive tasks. Training this domain may help automate the cognitive components of walking, and future neuroimaging studies would help elucidate the pathways by which speed of processing training confers benefits by those at risk for falls.

There are multiple possible explanations for the nonsignificant results in the full sample. First, our sample included a relatively low number of falls reported across the 10-year period. Our sample had a low occurrence of falls at baseline and across all follow-up periods (see Table 1; Supplementary Materials Table S1). For example, 16.40% of our full baseline sample reported a fall in the previous two months compared to approximately one-third of all older adults reporting a fall per year [34]. Second, although recall periods for falls are not consistent across studies, the current reference period included recalling falls within the past two months at baseline, annuals 1–3, 5, and 10. This likely failed to capture a significant number of falls and more heavily favored those individuals experiencing more frequent falls, which would likely be captured within the two months prior to assessment. Both issues are further demonstrated in the relatively small subsample of those receiving benefits, being those who were at greater risk of a future fall and receiving speed of processing training. Future work should continue to extend this work to less mobile older adults, particularly those with mobility limitations or Parkinson’s disease, to examine the effectiveness of cognitive training on falls for other at-risk populations. Additionally, future work should include better and more frequent assessments of falls to better capture this outcome.

Notably, poorer baseline Turn 360 performance consistently predicted the likelihood of a future fall across all analyses, except the high-risk subsample. While lower limb function and balance are associated with falls, poorer function does not always predict falling [35]. Most surprising was the lack of association between a fall occurrence and the demographic factors and cognitive status, particularly with age and MMSE. In addition to the low number of reported falls, it may have been due to the inclusion criteria why these two variables were not associated with falls. The likelihood of experiencing a fall typically increases across older adulthood [34], and poorer performance on the MMSE is associated with an increased fall risk [36]. In this sample, those who were not healthy at baseline or scoring a 22 or below on the MMSE were excluded, which may have resulted in a biased sample of healthy oldest-old adults, as well as those who are fairly homogenous (i.e., high performing) on their baseline MMSE score. The discrepancy between the current findings with the larger literature may be more reflective of the study inclusion criteria rather than a true lack of association between age, cognitive status, and falls in older adulthood.

There are some limitations of the current study. One major limitation is the fall assessment. While reducing the recall period of a fall to the prior two months rather than other windows (e.g., falls in the past year), there is a general tendency to underreport falls [34]. There were also long periods of time where individuals may have experienced a fall without reporting it (e.g., in the time between the 5- and 10-year assessments). With the advent of ecological assessment since the original ACTIVE trial’s inception, it is possible to reduce the recall period to the within-day level to reduce recall burden. Future studies may wish to consider examining the impact of cognitive training on falls using more frequent fall assessments to ensure that a fall event is not missed. As discussed above, a second major limitation toward generalizability is the general health of the ACTIVE participants. Not only was there a relatively low prevalence of falls at baseline, but the sample was intentionally selected to perform well on a cognitive status screener (i.e., MMSE). While the ACTIVE trial was adequately suited to examine high-functioning older adults, there was less power to determine the effect of different cognitive training programs on at-risk older adults. Future work should continue examining cognitive training programs in reducing falls for older adults with poorer mobility and lower limb function, as they may be those likeliest to receive benefits from training, since they are likelier to experience a fall in general. A third limitation to highlight was the inability to control for changes in other activities that might have impacted falls over the 10-year follow-up period. There are many factors that can contribute to falling that were not assessed in the ACTIVE trial, such as physical activity engagement. It is possible that engaging in these activities, rather than speed of processing training, were responsible for the reduction in falls. It is possible that mere participation in a behavioral intervention for maintaining cognitive function may prompt behavioral change elsewhere, and future research should examine the extent to which this occurs in intervention trials regardless of the intervention itself. Lastly, these analyses did not control for multiple comparisons. Although our analyses support prior work that individuals with poorer physical function may derive the greatest benefit from speed of processing training, this should be examined with a larger sample of high-risk fallers.

Taken together, this study found no evidence of the effectiveness of three cognitive training programs in reducing the time to a reported fall over 10 years across low-risk older adults, but previous fallers were approximately 30% less likely to experience a fall if they had speed of processing training. Engaging in speed-based cognitive exercises such as those available through BrainHQ (the commercially available training program based on ACTIVE’s speed of processing training) may be useful for older adults who have previously fallen, especially if they exhibit slow gait. Additionally, physical-based interventions to reduce falls may be further enhanced by incorporating cognitive speed of processing training. However, future work is needed to replicate these findings in a larger sample of at-risk older adults.

## Figures and Tables

**Table 1:** Baseline characteristics and group differences by baseline experience of a fall.

	No-Contact Control (*n* = 698)	Speed of Processing Training (*n* = 702)	Memory Training (*n* = 703)	Reasoning Training (*n* = 699)	Full Sample (*n* = 2802)	Differences by Baseline Experience of a Fall (*n* = 2793) †
Fall Experience PostBaseline, *n* (*%*) yes	251 (35.96%)	251 (35.75%)	228 (32.43%)	258 (36.91%)	983 (35.08%)	-
Baseline Fall, *n* (*%*) yes	114 (16.40%)	125 (17.86%)	99 (14.10%)	104 (14.88%)	442 (15.78%) †	-
Turn 360,number of steps, *M* (*SD*)	6.95 (2.11)	6.90 (1.97)	6.89 (1.99)	7.01 (2.25)	6.94 (2.08)	No Fall: 6.85 (1.99) Fall: 7.32 (2.21) ***
Age (years), *M* (*SD*)	74.05 (6.05)	73.42 (5.78)	73.53 (6.02)	73.53 (5.76)	73.63 (5.90)	No Fall: 73.59 (5.86) Fall: 73.78 (5.88)
Women, *n* (*%*)	514 (73.64%)	538 (76.64%)	537 (76.39%)	537 (76.82%)	2121 (75.70%)	No Fall: 1790/2351 (76.14%) Fall: 328/442 (74.09%)
White, *n* (*%*)	498 (71.35%)	518 (73.79%)	519 (73.83%)	503 (71.96%)	2038 (72.73%)	No Fall: 1697/2351 (72.22%) Fall: 332/442 (75.11%)
Education (years), *M* (*SD*)	13.37 (2.70)	13.65 (2.68)	13.59 (2.73)	13.50 (2.69)	13.53 (2.08)	No Fall: 13.51 (2.69) Fall: 13.61 (2.78)
MMSE (total score), *M* (*SD*)	27.27 (2.00)	27.43 (1.97)	27.29 (2.05)	27.26 (2.01)	27.31 (2.01)	No Fall: 27.34 (1.99) Fall: 27.19 (2.08)

Note. † Nine participants were missing baseline experience of a fall. *** *p* < 0.001.

**Table 2:** ITT Cox regression results for future falls among the full sample.

	**HR (95% CI)**	***p*-Value**
Randomized to Training		
Speed of training vs. control group	0.91 (0.76, 1.09)	0.32
Memory training vs. control group	0.88 (0.74, 1.06)	0.19
Reasoning training vs. control group	1.00 (0.83, 1.19)	0.96
Turn 360	1.11 (1.07, 1.14)	<0.001
Randomized to Booster		
Speed of training vs. control group	0.97 (0.76, 1.24)	0.82
Memory training vs. control group	0.93 (0.71, 1.22)	0.60
Reasoning training vs. control group	0.96 (0.75, 1.23)	0.76
Turn 360	1.07 (1.03, 1.12)	0.001

## Data Availability

The data used for these analyses are publicly available through the Inter-University Consortium for Political and Social Research (https://www.icpsr.umich.edu/icpsrweb/NACDA/studies/36036; accessed on 1 January 2019). Syntax or additional study materials are available upon request by contacting the corresponding author: Briana N. Sprague). The authors attest that these data have not been publicly presented elsewhere.

## Supplementary Materials

The following supporting information can be downloaded at: https://www.mdpi.com/article/10.3390/ijerph20064941/s1, Table S1: Reported falls per follow-up assessment, *n* (%).

## References

[1] National Center for Injury Prevention and Control (2015). 10 Leading Causes of Nonfatal Injury.

[2] Ensrud K.E. (2013). Epidemiology of fracture risk with advancing age. J. Gerontol. Ser. A Biol. Sci. Med. Sci..

[3] Piccirillo J.F., Vlahiotis A., Barrett L.B., Flood K.L., Spitznagel E.L., Steyerberg E.W. (2008). The changing prevalence of comorbidity across the age spectrum. Crit. Rev. Oncol./Hematol..

[4] Jørgensen V., Forslund E.B., Opheim A., Franzén E., Wahman K., Hultling C., Seiger Å., Ståhle A., Stanghelle J.K., Roaldsen K.S. (2017). Falls and fear of falling predict future falls and related injuries in ambulatory individuals with spinal cord injury: A longitudinal observational study. J. Physiother..

[5] Murphy S.L., Dubin J.A., Gill T.M. (2003). The development of fear of falling among community-dwelling older women: Predisposing factors and subsequent fall events. J. Gerontol. Ser. A Biol. Sci. Med. Sci..

[6] Boyd R., Stevens J.A. (2009). Falls and fear of falling: Burden, beliefs and behaviours. Age Ageing.

[7] Sherrington C., Michaleff Z.A., Fairhall N., Paul S.S., Tiedemann A., Whitney J., Cumming R.G., Herbert R.D., Close J.C., Lord S.R. (2017). Exercise to prevent falls in older adults: An updated systematic review and meta-analysis. Br. J. Sport. Med..

[8] Gillespie L.D., Robertson M.C., Gillespie W.J., Sherrington C., Gates S., Clemson L., Lamb S.E. (2012). Interventions for preventing falls in older people living in the community. Cochrane Database Syst. Rev..

[9] de Labra C., Guimaraes-Pinheiro C., Maseda A., Lorenzo T., Millán-Calenti J.C. (2015). Effects of physical exercise interventions in frail older adults: A systematic review of randomized controlled trials. BMC Geriatr..

[10] Ross L.A., Sprague B.N., Phillips C.B., O’Connor M.L., Dodson J.E. (2018). The impact of three cognitive training interventions on older adults’ physical functioning across 5 years. J. Aging Health.

[11] Smith-Ray R.L., Hughes S.L., Prohaska T.R., Little D.M., Jurivich D.A., Hedeker D. (2013). Impact of cognitive training on balance and gait in older adults. J. Gerontol. Ser. B Psychol. Sci. Soc. Sci..

[12] Azadian E., Majlesi M., Jafarnezhadgero A.A. (2018). The effect of working memory intervention on the gait patterns of the elderly. J. Bodyw. Mov. Ther..

[13] Azadian E., Torbati H.R., Kakhki A.R., Farahpour N. (2016). The effect of dual task and executive training on pattern of gait in older adults with balance impairment: A randomized controlled trial. Arch. Gerontol. Geriatr..

[14] Li K.Z.H., Roudaia E., Lussier M., Bherer L., Leroux A., McKinley P.A. (2010). Benefits of cognitive dual-task training on balance performance in healthy older adults. J. Gerontol. Ser. A Biol. Sci. Med. Sci..

[15] Marusic U., Grosprêtre S. (2018). Non-physical approaches to counteract age-related functional deterioration: Applications for rehabilitation and neural mechanisms. Eur. J. Sport Sci..

[16] Marusic U., Kavcic V., Giordani B., Gerževič M., Meeusen R., Pišot R. (2015). Computerized spatial navigation training during 14 days of bed rest in healthy older adult men: Effect on gait performance. Psychol. Aging.

[17] Marusic U., Verghese J., Mahoney J.R. (2018). Cognitive-based interventions to improve mobility: A systematic review and meta-analysis. J. Am. Med. Dir. Assoc..

[18] Ng T.P., Feng L., Nyunt M.S.Z., Feng L., Niti M., Tan B.Y., Chan G., Khoo S.A., Chan S.M., Yap P. (2015). Nutritional, physical, cognitive, and combination interventions and frailty reversal among older adults: A randomized controlled trial. Am. J. Med..

[19] Smith-Ray R.L., Makowski-Woidan B., Hughes S.L. (2014). A randomized trial to measure the impact of a community-based cognitive training intervention on balance and gait in cognitively intact black older adults. Health Educ. Behav..

[20] Verghese J., Mahoney J., Ambrose A.F., Wang C., Holtzer R. (2010). Effect of cognitive remediation on gait in sedentary seniors. J. Gerontol. Ser. A Biol. Sci. Med. Sci..

[21] Ball K.K., Edwards J.D., Ross L.A. (2007). The impact of speed of processing training on cognitive and everyday functions. J. Gerontol. Ser. B Psychol. Sci. Soc. Sci..

[22] Ball K.K., Ross L.A., Roth D.L., Edwards J.D. (2013). Speed of processing training in the ACTIVE study: How much is needed and who benefits?. J. Aging Health.

[23] Edwards J.D., Myers C., Ross L.A., Roenker D.L., Cissell G.M., McLaughlin A.M., Ball K.K. (2009). The longitudinal impact of cognitive 25speed of processing training on driving mobility. Gerontologist.

[24] Ross L.A., Edwards J.D., O’Connor M.L., Ball K.K., Wadley V.G., Vance D.E. (2016). The transfer of cognitive speed of processing training to older adults’ driving mobility across 5 years. J. Gerontol. Ser. B Psychol. Sci. Soc. Sci..

[25] Edwards J.D., Delahunt P.B., Mahncke H.W. (2009). Cognitive speed of processing training delays driving cessation. J. Gerontology. Ser. A Biol. Sci. Med. Sci..

[26] Ross L.A., Freed S.A., Edwards J.D., Phillips C.B., Ball K. (2017). The impact of three cognitive training programs on driving cessation across 10 years: A randomized controlled trial. Gerontologist.

[27] Edwards J.D., Ruva C.L., O’Brien J.L., Haley C.B., Lister J.J. (2013). An examination of mediators of the transfer of cognitive speed of processing training to everyday functional performance. Psychol. Aging.

[28] Jobe J.B., Smith D.M., Ball K., Tennstedt S.L., Marsiske M., Willis S.L., Rebok G.W., Morris J.N., Helmers K.F., Leveck M.D. (2001). ACTIVE: A cognitive intervention trial to promote independence in older adults. Control. Clin. Trials.

[29] Gill T.M., Williams C.S., Tinetti M.E. (1995). Assessing risk for the onset of functional dependence among older adults: The role of physical performance. J. Am. Geriatr. Soc..

[30] Steinhagen-Thiessen E., Borchelt M., Baltes P.B., Mayer K.U. (1999). Morbidity, Medication, and Functional Limitations in Very Old Age.

[31] Lipsitz L.A., Jonsson P.V., Kelley M.M., Koestner J.S. (1991). Causes and correlates of recurrent falls in ambulatory frail elderly. J. Gerontol. Ser. A Biol. Sci. Med. Sci..

[32] Sprague B.N., Phillips C.B., Ross L.A. (2019). Age-varying relationships between physical function and cognition in older adulthood. J. Gerontol. Ser. B Psychol. Sci. Soc. Sci..

[33] Folstein M.F., Folstein S.E., McHugh P.R. (1975). “Mini-mental state”: A practical method for grading the cognitive state of patients for the clinician. J. Psychiatr. Res..

[34] Masud T., Morris R.O. (2001). Epidemiology of falls. Age Ageing.

[35] Muir S.W., Berg K., Chesworth B., Klar N., Speechley M. (2010). Balance impairment as a risk factor for falls in community-dwelling older adults who are high functioning: A prospective study. Phys. Ther..

[36] Gleason C.E., Gangnon R.E., Fischer B.L., Mahoney J.E. (2009). Increased risk for falling associated with subtle cognitive impairment: Secondary analysis of a randomized clinical trial. Dement. Geriatr. Cogn. Disord..

