# Supplementary material for: Does Cognitive Training Reduce Falls across Ten Years?: Data from the ACTIVE Trial
Source: Int J Environ Res Public Health. 2023 Mar 11;20(6):4941. doi: 10.3390/ijerph20064941 (PMC10048914; doi:10.3390/ijerph20064941)
Supplement: Supplementary file 1 [file ijerph-20-04941-s001.zip › ijerph-2244590-supplementary.pdf]

**SUPPLEMENTARY MATERIALS**

Supplementary Table S1. Reported falls per follow-up assessment, *n* (%).

| | Full Sample | Control | Speed of Processing | Memory | Reasoning |
|---|---|---|---|---|---|
| Annual 1 | 317/2280 (13.90%) | 82/571 (14.36%) | 72/584 (12.33%) | 73/572 (12.76%) | 90/553 (16.27%) |
| Annual 2 | 316/2192 (14.42%) | 78/539 (14.47%) | 73/556 (13.13%) | 78/556 (14.03%) | 87/541 (16.08%) |
| Annual 3 | 281/2075 (13.54%) | 72/505 (14.26%) | 68/531 (12.81%) | 71/528 (13.45%) | 70/511 (13.70%) |
| Annual 5 | 290/1855 (15.63) | 75/447 (16.78%) | 78/479 (16.28%) | 71/463 (15.33%) | 66/466 (14.16%) |
| Annual 10 | 237/1212 (19.55) | 58/284 (20.42%) | 61/317 (19.24%) | 49/297 (16.50%) | 69/314 (21.97%) |

*Note*. Reported falls include participants who reported more than one fall across the intervention period. For example, if a participant in the control group experienced a fall at Annual 1 and again at Annual 5, they would contribute to both cells. Omnibus ($\chi^2$) tests indicated no group differences at any assessment.
